# Conservative Care for L3/L4 Spondylolisthesis With Severe Stenosis in the Setting of L5 Sacralization

**DOI:** 10.7759/cureus.115887

**Published:** 2026-09-07

**Authors:** Eric Chun-Pu Chu, Peiyi Kong

**Affiliations:** 1 Chiropractic and Physiotherapy Centre, New York Medical Group, Hong Kong, CHN

**Keywords:** conservative management, lumbar spinal stenosis, lumbosacral transitional vertebra, magnetic resonance imaging, neurogenic claudication, sacralization, spondylolisthesis

## Abstract

Lumbosacral transitional vertebrae may alter load transfer and are associated with degenerative changes at spinal levels cranial to the transitional segment. However, the relationship between complete L5 sacralization and pathology at L3-L4 remains uncertain. We report a 69-year-old woman with chronic low-back pain, lower-extremity symptoms, and walking intolerance in the setting of complete bilateral L5 sacralization, L3-L4 grade I anterolisthesis, severe central canal stenosis, and Modic type 1 endplate changes. Given her cardiovascular comorbidity, functional goals, and preference for nonoperative care, she underwent nine months of individualized multimodal conservative management that included low-force pelvic blocking, motorized flexion-distraction therapy, radial shockwave therapy applied to symptomatic soft tissues, home advice, and progressive exercise. At month nine, pain improved from 8/10 to 1/10, lower-extremity symptoms resolved, neural-tension tests were negative, and the patient returned to unrestricted restaurant work. Follow-up magnetic resonance imaging reported L3-L4 anterolisthesis of 0.53 cm compared with 0.65 cm at baseline, together with less apparent marrow edema and facet fluid. This case documents clinical improvement during multimodal conservative care in a patient with complex degenerative lumbar pathology and complete L5 sacralization. The observed imaging differences do not establish true slip reduction, identify the effective treatment component, or confirm a causal biomechanical mechanism. Prospective studies using standardized imaging acquisition, blinded repeated measurements, and comparison groups are required.

## Introduction

Lumbosacral transitional vertebrae are congenital spinal variations in which the lowest lumbar vertebra demonstrates partial or complete transitional features with the sacrum. Sacralization of L5 may reduce motion at the transitional level and has been associated with altered degeneration patterns at cranial mobile segments. The association between lumbosacral transitional vertebrae and low-back pain remains debated; however, pain and degenerative pathology may arise from the segment above the transition, the contralateral facet in unilateral cases, or the anomalous articulation itself [1].

In clinical practice, evaluating spondylolisthesis and segmental instability relies on upright weight-bearing lateral radiographs and dynamic flexion-extension views, which capture gravity-loaded translational alignment. While magnetic resonance imaging (MRI) provides detailed cross-sectional visualization of neural compression and marrow edema, supine positioning unloads the spine and introduces measurement variability. Consequently, defining true 'structural reduction' or anatomical slip correction requires standardized, weight-bearing imaging protocols with validated landmarking reliability beyond ordinary scanner positioning margins.

Observational studies suggest that sacralization may change the distribution of disc degeneration and spondylolisthesis to the cranial adjacent mobile segment, commonly L4-L5. In patients with L4 spondylolysis, sacralization has been associated with greater vertebral slip and more severe disc degeneration [2]. Recent studies of lumbosacral transitional vertebrae similarly report altered degenerative disease patterns at levels adjacent to the transitional vertebra, although these observations do not establish direct biomechanical causation or predict pathology at more cranial levels such as L3-L4 [3,4].

Lumbar spinal stenosis can produce low-back pain, lower-extremity symptoms, and neurogenic claudication. Guidelines and systematic reviews support individualized multimodal nonoperative management, including education, exercise, rehabilitation, and selected manual-therapy approaches, for many patients with lumbar spinal stenosis and neurogenic claudication. However, the comparative effectiveness of individual treatment components remains uncertain [5,6].

This case report describes serial clinical and magnetic resonance imaging findings during nine months of multimodal conservative care in a patient with complete L5 sacralization, L3-L4 grade I anterolisthesis, severe central canal stenosis, and Modic type 1 endplate changes. It also presents the hypothesis that reduced motion at the transitional lumbosacral level may have contributed to loading at cranial lumbar segments. This hypothesis was not directly tested and is not presented as a validated biomechanical mechanism.

## Case presentation

A 69-year-old woman working in a restaurant and with a history of competitive judo presented with a multi-year history of chronic low-back pain and recent symptom exacerbation. She described constant aching low-back pain rated 8/10 on a Numeric Rating Scale (NRS), with bilateral lower-extremity numbness and pulling pain involving the lateral thighs. Symptoms were aggravated by sit-to-stand transitions, prolonged standing, and lumbar extension. Her medical history included triple coronary artery bypass grafting, essential hypertension, and bilateral total knee arthroplasties.

On examination, she demonstrated an antalgic gait and painful restriction of lumbar movement. Slump and straight-leg-raise tests reproduced lower-extremity symptoms bilaterally at 40°. Lower-extremity motor testing was 4+/5 in the bilateral L4 and L5 myotomes, with pain reported during testing. Light-touch sensation was diminished in the right L3 and L4 dermatomes.

Weight-bearing lateral lumbar radiographs showed mild lumbar levoscoliosis, grade I L3-L4 anterolisthesis, multilevel disc-space narrowing, and anterior osteophytes. Magnetic resonance imaging demonstrated complete bilateral sacralization of L5, consistent with Castellvi type IIIb morphology, and grade I L3-L4 anterolisthesis reported as 0.65 cm (Figure 1A). Bilateral L3 and L4 pars defects were reported. At L3-L4, posterior disc pathology, right extraforaminal protrusion, facet hypertrophy, and ligamentum flavum thickening were associated with severe central canal stenosis, with an anteroposterior canal diameter reported as 0.56 cm. The radiology report also described crowding of the cauda equina nerve roots and right L3 exiting nerve-root compression. Additional findings included right L3-L4 facet arthropathy with marrow edema and joint fluid, together with Modic type 1 endplate changes at L3-L4.

**Figure 1:**
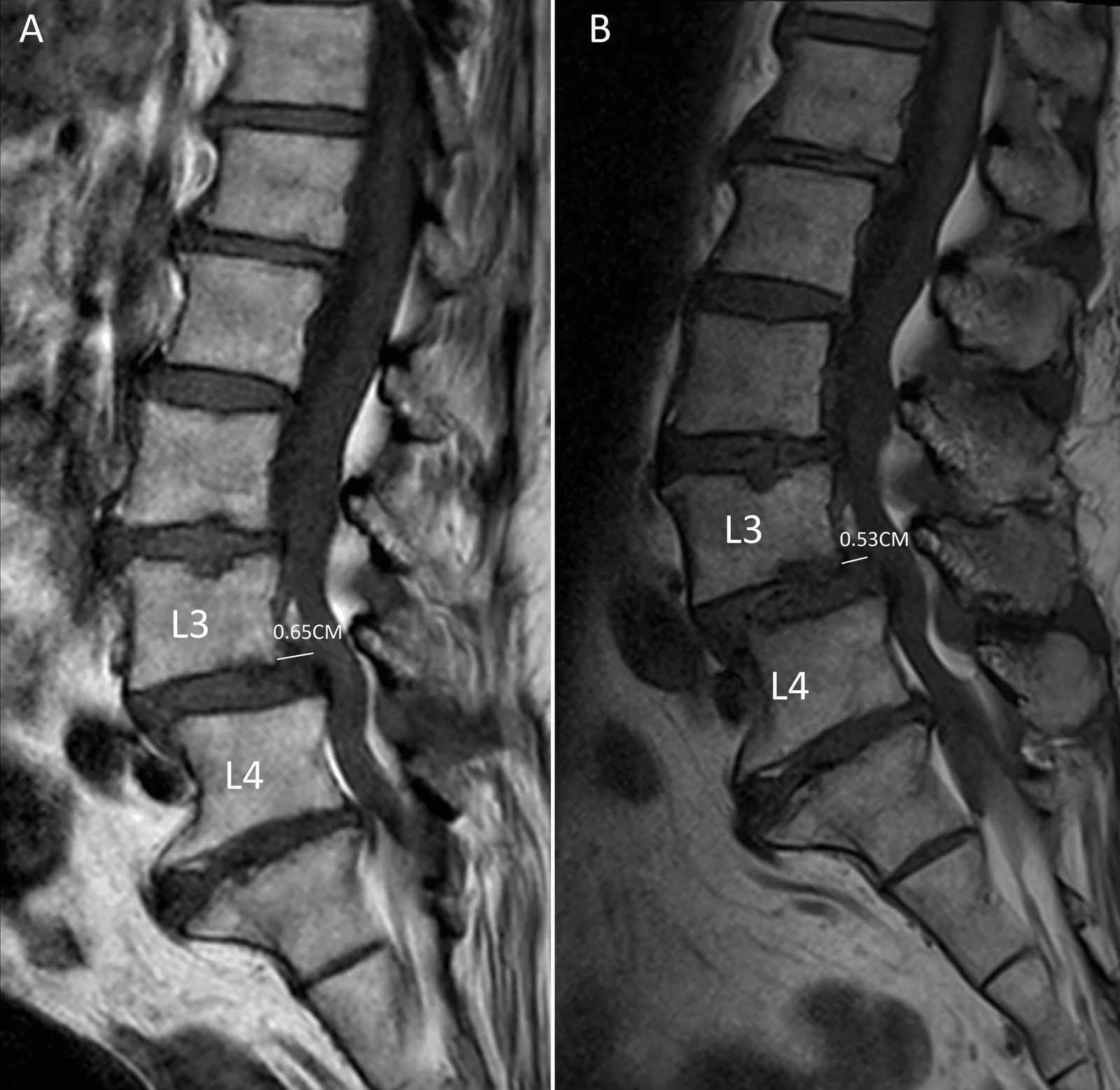
Serial lumbar magnetic resonance imaging. (A) Baseline sagittal MRI showing L3–L4 anterolisthesis (reported as 0.65 cm), severe canal stenosis, and Modic type 1 endplate signal changes. (B) Month-nine follow-up sagittal MRI (reported as 0.53 cm anterolisthesis). Note: The baseline and follow-up scans differ in slice selection and acquisition settings, and lack matched T2 fat-suppressed or STIR sequences. Apparent attenuation of marrow edema or joint fluid across non-identical, standard clinical sequences cannot establish true biological remodeling. STIR: short tau inversion recovery.

Therapeutic intervention

Given the patient's clinical complexity, history of coronary artery bypass grafting, and treatment preferences, an individualized multimodal conservative-care program was delivered over nine months, with 48 planned visits. 

Spinal manipulation was applied to the L3-L4 region. Sacro-Occipital Technique pelvic blocking was applied in prone and supine positions for 8-10 minutes per session. Motorized flexion-distraction therapy was delivered in a slow rhythmic manner at the L3-L4 region for 3-5 minutes per session. Radial shockwave therapy was applied weekly to symptomatic bilateral gluteus medius, piriformis, and lateral-thigh soft tissues at 1.8-2.2 bar, 2,000 shocks, and 8 Hz. Home management included heat application, activity and posture advice, omega-3 supplementation, and submaximal trunk-stabilization exercises.

During the second month, the patient experienced a fall onto the lower back and gluteal region, followed by bruising, pain exacerbation, and a one-week hospital observation stay. After discharge, the intervention was modified temporarily. Flexion-distraction was performed with knee flexion and a pillow between the knees for comfort. From month three onward, progressive gluteal activation, piriformis stretching, and low-load trunk-stabilization exercises were reintroduced according to symptoms and tolerance.

The intervention was multimodal and adapted over time. It was not designed to determine the independent effectiveness or mechanism of pelvic blocking, flexion-distraction, shockwave therapy, exercise, home advice, or other concurrent factors.

Outcomes and follow-up

The patient reported progressive improvement over nine months. At month nine, low-back pain decreased from 8/10 to 1/10. She reported resolution of lower-extremity numbness and pulling pain. Slump and straight-leg-raise tests were negative to 80° bilaterally. She progressed from requiring assistance during sit-to-stand transitions to performing them independently and returned to full restaurant shifts without self-reported physical limitation.

A follow-up MRI was obtained at month nine. The reported L3-L4 anterolisthesis measurement was 0.53 cm compared with 0.65 cm at baseline (Figure 1B). The follow-up radiology report described less apparent endplate marrow edema and facet fluid. However, these observations are subject to substantial technical limitations. The serial MRIs were obtained as part of standard clinical care and lack matched T2-weighted fat-suppressed or short tau inversion recovery (STIR) sequences. 

The 1.2 mm difference between reported measurements is an imaging observation. It cannot, by itself, establish true reduction of vertebral translation because MRI measurements may vary with patient positioning, slice selection, scanner protocol, image quality, landmark selection, and reader interpretation. Similarly, Modic changes may evolve over time independent of treatment, and their temporal association with symptom change does not establish treatment causality [7,8].

## Discussion

This case describes marked improvement in pain, lower-extremity symptoms, functional transitions, neural-tension findings, and work participation during nine months of individualized multimodal conservative care in a patient with complete L5 sacralization, L3-L4 anterolisthesis, severe central canal stenosis, and Modic type 1 changes.

Lumbosacral transitional vertebrae may alter loading at cranial mobile segments. The segment immediately above a sacralized L5, frequently L4-L5, has been most consistently implicated in adjacent degeneration or altered spondylolisthesis distribution [1-4]. In the present patient, L3-L4 pathology coexisted with complete L5 sacralization and multilevel degenerative change. It is possible that reduced mobility at the transitional level, degeneration at adjacent segments, prior athletic exposure, age-related degeneration, facet arthropathy, and pars defects contributed to the L3-L4 pathology. The relative contribution of these factors cannot be determined from a single case.

The proposed kinetic lever-arm explanation is hypothesis-generating: this case did not measure segmental motion, loading, muscle activity, or other biomechanical variables needed to test it. The case does not demonstrate that L5 sacralization created a specific extended moment arm or that it caused the L3-L4 slip, pars defects, endplate changes, or stenosis.

Evidence supports a multimodal, individualized conservative approach for some patients with lumbar spinal stenosis and neurogenic claudication. Updated systematic review evidence indicates that manual therapy and exercise, with or without education, may improve symptoms, function, and walking capacity for selected patients with lumbar spinal stenosis, although evidence for many individual nonoperative treatments remains limited [5,6]. Small studies and pilot investigations have evaluated flexion-distraction approaches for lumbar spinal stenosis, but these studies do not establish that flexion-distraction changes canal dimensions, reduces vertebral translation, reverses disc prolapse, or provides superior outcomes compared with other interventions [9-11].

The difference in reported L3-L4 translation on follow-up MRI should be interpreted cautiously. Upon independent review, observers may perceive the translation as unchanged or even subtly altered depending on slice registration, sagittal contour, and chosen measurement landmarks. Supine MRI eliminates axial gravity loading, which can cause spontaneous recumbent reduction or understate true standing instability compared with erect posture. Consequently, subtle translation differences between non-weight-bearing scans may simply reflect positional unweighting rather than anatomical remodeling, underscoring why routine upright weight-bearing radiographs (and dynamic flexion-extension views) remain indispensable for definitive assessments of translational stability. A 1.2 mm difference falls well within ordinary measurement and positioning variability unless standardized acquisition protocols and blinded reliability analyses are employed. The apparent attenuation of Modic type 1 changes should similarly not be attributed to treatment. Longitudinal studies show that Modic findings can change in type and extent over time, including transitions from type 1 to type 2 and, less commonly, apparent resolution [7,8].

This case report has several methodological limitations regarding clinical reproducibility and patient profiling. Although the clinical history, pain scores (8/10 NRS), dermatomal deficits, and manual myotomal grades (4+/5) provide a general clinical picture, the assessment relied on subjective functional reporting rather than validated spine-specific disability indices (e.g., Oswestry Disability Index) or quantitative calibrated dynamometry (e.g., isometric trunk and hip testing). Furthermore, baseline upright weight-bearing and flexion-extension stress radiographs were not performed. Clinicians seeking to identify comparable patient presentations should note that future studies require standardized quantitative functional metrics and dynamic weight-bearing imaging to establish true clinical baseline equivalence. The proposed biomechanical interpretation is hypothesis-generating and requires evaluation in prospective studies using standardized weight-bearing and supine imaging, blinded repeated measurements, validated clinical outcomes, and comparator groups.

## Conclusions

This case report documents substantial clinical and functional improvement during nine months of multimodal conservative care in a patient with complete L5 sacralization, L3-L4 grade I anterolisthesis, and severe lumbar stenosis. A difference in MRI-reported L3-L4 translation and attenuation of selected inflammatory imaging findings was observed at follow-up. These observations do not establish true structural slip reduction, anatomical remodeling, treatment efficacy, or a causal L3-L4 kinetic lever-arm mechanism. The proposed biomechanical interpretation is hypothesis-generating and requires evaluation in prospective studies using standardized weight-bearing and supine imaging, blinded repeated measurements, validated clinical outcomes, and comparator groups.
